# Histological Characteristics and Management of Hepatitis on Immune Checkpoint Inhibitors: A Retrospective Descriptive Study

**DOI:** 10.3390/jcm12113751

**Published:** 2023-05-29

**Authors:** Lucia Parlati, Kennie Marcin, Benoit Terris, Anaïs Vallet-Pichard, Marion Corouge, Clémence Hollande, Philippe Sogni, Vincent Mallet, Stanislas Pol

**Affiliations:** 1 Faculty of Medicine, Université Paris Cité, Cochin Site, 24 rue du Faubourg Saint-Jacques, F-75006 Paris, France; kennie.marcin@gmail.com (K.M.); benoit.terris@aphp.fr (B.T.); philippe.sogni@aphp.fr (P.S.); vincent.mallet@aphp.fr (V.M.); stanislas.pol@aphp.fr (S.P.); 2 AP-HP. Centre, Groupe Hospitalier Cochin Port Royal, DMU Cancérologie et Spécialités Médico-Chirurgicales, Service d’Hépatologie, F-75014 Paris, France; anais.vallet-pichard@aphp.fr (A.V.-P.); marion.corouge@aphp.fr (M.C.); 3 AP-HP. Centre, Groupe Hospitalier Cochin Port Royal, DMU IMAGINA, Service de Pathologie, F-75014 Paris, France; 4 Liver Cancer Unit, AP-HP, Hôpital Beaujon, F-92110 Clichy, France; clemence.hollande@aphp.fr

**Keywords:** immune checkpoint inhibitors (ICI), ICI-related drug-induced liver injury (ICI-DILI), hepatitis, histology of ICI-DILI

## Abstract

Background and aims: the side effects of immune checkpoint inhibitors (ICI) pose a problem for the clinical management of cancer patients. There is a lack of knowledge of the value of liver biopsy in patients with ICI-related drug-induced liver injury (ICI-DILI). The aim of this study was to explore the impact of liver biopsy on clinical management and response to corticosteroids, according to histological findings. Methods: We conducted a retrospective, single-center study to evaluate the biochemical, histological and clinical data of 35 patients with ICI-DILI between 2015 and 2021 in a university hospital in France. Results: Of the 35 patients with ICI-DILI (median [interquartile range] age 62 [48–73] years, 40% males) studied, 20 underwent a liver biopsy. There was no difference in the management of ICI-DILI according to liver biopsy in terms of ICI withdrawal, reduction or rechallenge. According to the histological profile, patients with toxic and granulomatous profiles had a better response to corticosteroids, while patients with cholangitic lesions had the worst response. Conclusion: In ICI-DILI, liver biopsy must not delay patient care but may be useful in identifying patients with a cholangitic profile who have a poorer response to corticosteroids.

## 1. Introduction

Since their approval in 2010, immune checkpoint inhibitors (ICI) have revolutionized the management and outcome of patients with advanced cancer [1]. Their mechanism of action consists in lifting the immune inhibition generated by tumor cells on key elements of the immune cascade, such as PD-1 (programmed cell death 1) and CTLA-4 (cytotoxic T-lymphocyte antigen 4) located on the immune cells. Their efficacy, largely proven, may be limited by their immuno-mediated side effects, which may affect every organ, including the liver [2].

Several mechanisms have been explored to explain ICI toxicity; however, the specific pathogenetic mechanisms underlying ICI hepatitis are still not completely defined.

The first possible mechanism is direct immune toxicity. PD-1 and PD-L1 are expressed on healthy tissue cells, suggesting that cytotoxicity under ICI is possible by the activation of complement against the “*self non-tumor cells*” [3].

The depletion of regulatory T cells (Treg) has also been suggested to play a role in the development of ICI toxicities, as Treg cells are essential for maintaining peripheral tolerance. CTLA-4 is constitutively expressed on Treg cells and its blockade can affect Treg cell number and function. In fact, a reduction in serum Treg cell count and an increased T-cell-effector-to-Treg-cell ratio has been observed in patients treated with ipilimumab. Moreover, preclinical models have shown a negative correlation between the number of peripheral Treg cells and ICI toxicities [4].

Finally, B cells also appear to play a role in the development of ICI toxicity, as early changes in B cell populations, with the presence of autoreactive lymphocytes, can be seen in patients treated with ICI who develop levels of toxicity grade greater than 3 [5].

Acute ICI- related drug-induced liver injury (ICI-DILI) has been reported to occur in 1.0–4.5% of treated patients, and may be severe in 0.14–4.8 % of cases [2,6].

The diagnosis of ICI-DILI is based on a combination of clinical, biochemical, virological and radiological findings, to rule out all the differential diagnoses of acute hepatitis [7,8,9].

The role of liver biopsy in the diagnosis of ICI-DILI is unclear, and guidelines vary widely. According to most experts, liver biopsy is generally not necessary for the routine diagnosis and management of ICI-DILI, but may be useful for patients who do not respond to conventional treatment, or in whom the diagnosis is questionable due to an atypical presentation or an unusual clinical course. In such cases, liver histology can provide important and useful information about the type of lesion and its severity and can help identify other causes of hepatitis [10].

Management of ICI-DILI depends on the severity of the hepatitis. It is based on the withdrawal or dose reduction of the immunotherapy according to the severity of ICI-DILI, the introduction of corticosteroids or immunosuppressive therapy, and, eventually, the reintroduction of immunotherapy (the same or another molecule). Many studies reported that liver tests can improve rapidly after simple discontinuation of ICI, without introducing corticosteroids. Recently, it has been proposed that the introduction of corticosteroids should be limited to patients with severe liver toxicity (total bilirubin > 2.5 mg/dl, INR (International Normalized Ratio) > 1.5 or a constant worsening of the hepatitis after ICI withdrawal). This might avoid high doses of corticosteroids, often unnecessary, with their several side effects. Ursodeoxycholic acid (UDCA) can be added to patients presenting with biochemical cholestasis [2].

The aim of our study was to explore the impact of liver biopsy on the management of patients with ICI-DILI, in order to clarify the interest of histology in the diagnostic strategy. We also investigate the role of corticosteroids in the management of ICI hepatitis according to the histological profile of ICI-DILI.

## 2. Materials and Methods

### 2.1. Patients

We conducted a monocentric, retrospective study that included 35 consecutive patients with a confirmed diagnosis of ICI-DILI at Cochin Hospital-APHP, between 2015 and 2021. Twenty patients underwent a liver biopsy.

Patients in this study received ICI, either as monotherapy with an anti-PD-1, (Nivolumab or Pembrolizumab) or as combination therapy with an anti-PD1 and an anti-CTLA-4 (Ipilimumab).

Patients received ICI every 4 weeks on average (2 to 6 weeks); the treatment cycle at which acute hepatitis occurred was recorded.

The diagnosis and severity of ICI-DILI was defined according to the Common Terminology Criteria for Adverse Events (CTCAEv.5.0) guidelines [11].

Clinical characteristics such as age, sex, type and stage of cancer, history of chemotherapy or radiotherapy, line of treatment and common risk factors for liver disease such as alcohol consumption and metabolic syndrome were recorded.

All patients were questioned, to exclude the use of potentially hepatotoxic drugs. An exhaustive etiological workup was performed to rule out the different etiologies of acute hepatitis, including viral serologies (Hepatitis A virus (HAV), Hepatitis B virus (HBV), Hepatitis C virus (HCV), Hepatitis E virus (HEV), Herpes Simplex virus (HSV), Cytomegalovirus (CMV), Epstein–Barr virus (EBV) or Varicella zoster virus (VZV) hepatitis), and an autoimmune workup with antinuclear antibodies, anti-smooth muscle antibodies, anti-mitochondrial antibodies and liver/kidney microsomal antibodies type 1. All patients underwent liver imaging (ultrasonography or contrast-enhanced computed tomography) to rule out vascular or biliary etiology or the progression of secondary liver lesions.

The cytolytic or cholestatic biochemical profile was assessed by the R-factor (according to the American College of Gastroenterology). Cytolytic damage was defined by R-factor > 5 or ALT ≥ 5-fold above the upper limit of normal (ULN), while cholestatic damage was assessed by R-factor < 2 or ALP ≥ 2-fold above the upper limit of normal (ULN); mixed damage was defined by intermediate values.

The Roussel Uclaf Causality Assessment Method (RUCAM) was used by an expert hepatologist (LP) to assess the causal relationship between immunotherapy and ICI-DILI [12]. The interpretation of the final score was as follows: <0, drug is ‘excluded’ as a cause; 1–2, ‘unlikely’; 3–5, ‘possible’; 6–8, ‘probable’; and >8, ‘highly probable’.

We also recorded an ICI rechallenge, including the regimen used, any recurrence of hepatotoxicity, its latency, and management.

We finally documented overall survival rates and oncological disease progression after ICI-DILI.

The Institutional Review Board of Assistance Publique-Hôpitaux de Paris (CLEP-CCH) approved the study, and all patients’ data were anonymized (Ethic Committee Name: Comité Local d’Éthique pour les publications de l’hôpital Cochin (CLEP); Approval Code: AAA-2017-06007; Approval Date: 20 November 2017). The study protocol conforms to the ethical guidelines of the 1975 Declaration of Helsinki (6th revision, 2008).

### 2.2. Histological Evaluation

Patients were biopsied after the exclusion of other causes of acute hepatitis. The biopsy was performed as soon as ICI-DILI was suspected (within 15 days). Patients underwent a liver biopsy in order to confirm the diagnosis of ICI-DILI, to eliminate other causes of hepatitis and, for some, to decide whether to introduce corticosteroids. Histological analysis was performed by the same pathologist (BT) in an open setting (based on the medical record).

The histological criteria described were: inflammation localization, inflammation density and cell type (plasma cells, eosinophils, lymphocytes, neutrophils), liver fibrosis, presence and degree of necrosis, steatosis, the presence of cholangitic (cholangitis, ductopenia, etc.) or endothelial damage and the presence of tumor cells. An immunohistochemical study was not performed as a first-line analysis.

In this study, we classify liver toxicity according to histological findings, as follows:

- Acute granulomatous hepatitis, defined by the presence of granulomas;
- Acute hepatitis with an autoimmune profile, defined by the presence of plasma cells;
- Acute hepatitis with a toxic profile, defined by the presence of eosinophilic polynuclear cells.

Acute hepatitis was defined as “mixed” when plasma cells and eosinophils were found on histology, without granulomas. Patients without a defined histological profile did not present any of these characteristics, only inflammation and necrosis. A cholangitic profile was assigned when we recorded bile duct lesions (cholangitis, ductopenia, etc.). We did not observe any ICI-related liver vascular disease in our cohort.

No severe post-liver-biopsy complications were observed in our study.

### 2.3. Therapeutic Response

In patients who received treatment for ICI-DILI (withdrawal or reduction of immunotherapy, introduction of corticosteroids), response to therapy was defined as an improvement in liver tests of less than or equal to CTCAE v.5.0 grade 1, according to the American Society of Clinical Oncology (ASCO) recommendations [7].

A second biopsy was not performed to evaluate the histological evolution of hepatitis.

### 2.4. Statistical Analysis

Continuous variables were expressed as medians and interquartile range, while categorical variables were expressed as number of patients and percentages. Parametric and nonparametric tests were performed when appropriate. The Mann–Whitney U test was used for continuous variables. The chi2 test with Fisher correction was used for categorical variables. A *p* value of <0.05 was considered statistically significant. For statistical analysis, we used the IBM SPSS Statistics for Windows program, Version 21.0.

## 3. Results

### 3.1. Patients’ Characteristics according to Liver Biopsy

**Figure 1:**
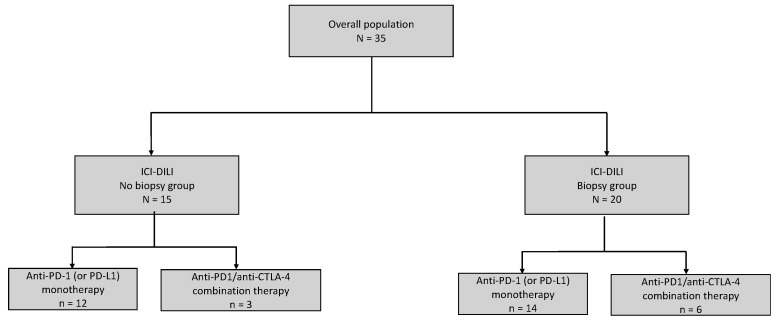
Flowchart of the study population.

Out of the 35 patients studied, 20 patients underwent liver biopsy (Biopsy group = B group). A total of 14 (70%) received anti-PD-1 monotherapy and 6 (30%) received anti-PD-1/anti-CTLA-4 combination therapy. Among the 15 patients who did not undergo liver biopsy (No biopsy group = NB group), 12 (80%) received anti-PD-1 monotherapy and 3 received anti-PD-1/anti-CTLA-4 combination therapy. (Figure 1).

There were 40% of male patients in both groups. The mean age was 59 (IQR, 49–65) and 69 (45–77) years in the B and NB groups, respectively; nobody had pre-existing liver disease or liver-function-test abnormalities before ICI-DILI. Three patients (15%) had liver metastases in the B group and two (13%) in the NB group. For these patients, metastasis progression was ruled out at diagnosis of ICI-DILI.

In the B group, 10 patients (50%) received ICI for melanoma, 7 patients (35%) for non-small-cell lung carcinoma, and 3 patients (15%) for another neoplasia. In the NB group, 9 patients (60%) received ICI for melanoma, 3 patients (20%) for non-small-cell lung carcinoma, 1 patient (7%) for lymphoma and 2 patients (13%) for another cancer.

There was no difference in terms of smoking habits, alcohol use, history of standard chemotherapy, or ICI regimen between the two groups. The patients’ characteristics according to the liver biopsy are detailed in Table 1.

If compared to NB group patients, B group patients had higher Alanine amino transferase (ALT) levels, of 642 U/L (123–796) vs. 154 U/L (95% CI 57–481) (*p* = 0.022), and a trend to higher Aspartate aminotransferase (AST) levels, of 281 (80–550) vs. 119 (51–207) (*p* = 0.059) (Table 1).

The R-factor supported a cytolytic profile in B group patients compared to NB group patients (*p* = 0.004).

No patient presented severe liver injury in our cohort.

There was no significative difference in RUCAM score between the two groups; it was 7.5 (6–8.7) in the B group and 7 (5.5–9.5) in the NB group.

The time for normalization of transaminases was 49 days in the NB group, and 60 days in the B group (*p* = 0.205).

There was no difference in the management of ICI-DILI according to liver biopsy in terms of ICI withdrawal, reduction or rechallenge; however, the introduction of corticosteroids (1–2 mg/kg/day) tended to be more frequent in B group patients than in NB group patients (14 (70%) vs. 6 (40%) patients, respectively (*p* = 0.080)). We finally observed a significant correlation between liver necrosis and ALT grade (*p* = 0.012).

### 3.2. Clinical and Histological Characteristics by ICI Type

Patients receiving anti PD-1/anti-CTLA-4 combination therapy were younger (*p* = 0.043) and exclusively treated for melanoma (*p* = 0.003), when compared to patients receiving anti PD-1 monotherapy.

There were no statistically significant differences between patients receiving anti-PD-1 monotherapy or anti-PD-1/anti-CTLA-4 combination therapy in terms of sex, number of treatments before the occurrence of ICI-DILI, previous chemotherapy, history of other adverse events regarding ICI, biological severity of ICI-DILI and introduction of corticosteroids (Table 2). We did not observe any difference in the R-factor or RUCAM score between the two groups.

However, we observed a longer time until the liver-function test resolution in patients receiving combination therapy when compared to patients receiving monotherapy (120 (75–150) days vs. 49 (37–67) days) (*p* = 0.007).

Patients receiving anti-PD-1 monotherapy more frequently had a toxic histological profile (36 % vs. 17 %) or no defined histological profile (36% vs. 17%), while half of the patients treated with the combination therapy had a granulomatous profile. When considering histological severity, we observed more frequently minimal necrosis in patients treated with monotherapy, while mild-to-severe necrosis was more frequent in patients treated with combination therapy (Table 2 and Table S1). Even if not statistically significant, we observed more cholangitis (67% vs. 36%, *p* = 0.509) and liver fibrosis (50% vs. 21%, *p* = 0.070) in patients receiving combination treatment as compared to anti PD-1 monotherapy.

ICI therapy had no impact on the decision of ICI withdrawal or rechallenge, but clinicians decided more often to carry out ICI dose reduction for patients treated with combination therapy (33% vs. 4%, *p* = 0.021).

**Figure 2:**
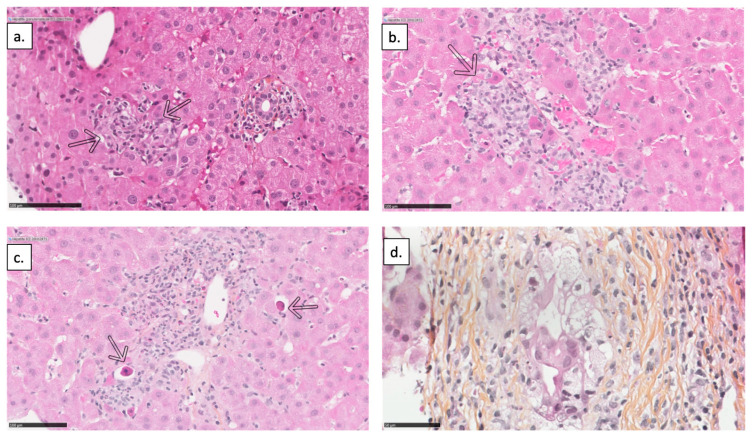
Histological findings of ICI-DILI. (**a**) Intra-lobular epithelioid microgranuloma (arrows); (**b**) Centro-lobular epithelioid microgranuloma; (**c**) Portal space containing a polymorphic inflammatory infiltrate with some eosinophilic polynuclear cells causing periportal hepatocellular necrosis (arrows) in favor of a toxic hepatitis; (**d**) An interlobular bile duct showing important cytologic damage to its epithelium.

Figure 2a,b represent a histological slice of granulomatous hepatitis, Figure 2c represents that of toxic hepatitis, and Figure 2d represents that of cholangitic lesion.

### 3.3. Response to Corticosteroids according to Histological and Biological Profile

**Figure 3:**
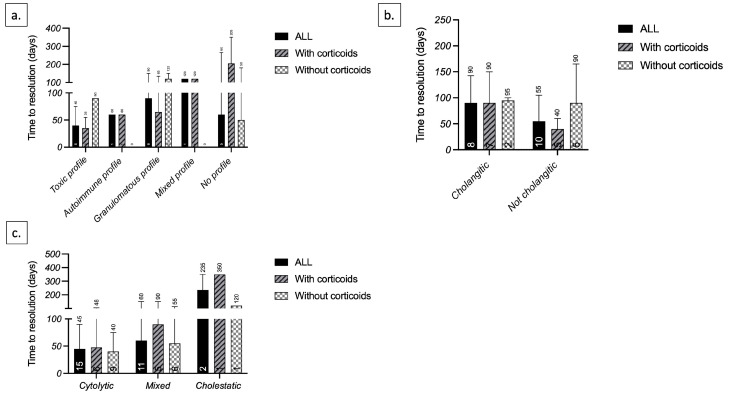
Time until liver-function test normalization according to (**a**) histological profile; (**b**) histological cholangitic lesions; and (**c**) biochemical profile (R-factor).

According to the histological profile, toxic and granulomatous profiles had a better response to corticosteroids; the median time for liver-function test resolution was 35 days and 90 days with and without corticosteroids, respectively, for the toxic profile, and 65 days and 120 days with and without corticosteroids, respectively, for the granulomatous profile. Patients without a defined histological profile had the worst response to corticosteroids; the median time for liver-function test resolution for patients without a defined profile was 205 days and 50 days with and without corticosteroids, respectively (Figure 3a).

We also observed a better response to corticosteroids in patients without cholangitic lesions; the median time for liver test resolution for these patients was 40 days and 90 days with and without corticosteroids, respectively (Figure 3b).

Finally, when considering the R-factor, patients with a mixed and cholestatic biological profile had a worst response to corticosteroids, while we did not observe any difference for the cytolytic profile (Figure 3c).

### 3.4. Management of ICI-DILI

According to international guidelines, management of ICI-DILI was based on the reduction of ICI in four patients (two in the NB group and two in the B group) or on ICI withdrawal in 87% of patients in the NB group and in 95% of patients in the B group (*p* = 0.390). Patients with ICI dose reduction were all treated for stage 4 melanoma, and two of them had grade 1–2 ICI-DILI.

Corticosteroids were administered to six (40%) patients of the NB group and to fourteen (70%) patients of the B group (*p* = 0.080); thirteen (50%) patients received anti-PD1 monotherapy and seven (78 %) the combination therapy. Finally, corticosteroids were given more often to patients with evidence of necrosis at liver histology (60% vs. 33%) and with more severe hepatitis (75 % vs. 53% of grade 3–4 ALT elevation) (*p* = 0.095).

According to the histological features:

- Intense necrosis was observed in seven (35%) patients receiving corticosteroids and in no patient not treated with corticosteroids (*p* = 0.036).
- Granulomatous lesions were observed in four (20%) patients receiving corticosteroids and in two (13%) patients not treated with corticosteroids (*p* = 0.913).
- Toxic profile was observed in five (25%) patients receiving corticosteroids and in one (7%) patient not treated with corticosteroids (*p* = 0.406).
- Autoimmune profile was observed in one (5%) patient receiving corticosteroids and in no patient not treated with corticosteroids (*p* = 0.497) (Table 3).

### 3.5. Characteristics of Patients Retreated with ICI

An ICI rechallenge was carried out in eight (22%) patients of the study group and did not result in recurrence of liver toxicity. Four (50%) patients belonged to the B group; five (63%) were treated with ICI monotherapy and three (38%) with combination therapy.

At ICI-DILI, hepatitis was biologically severe (grade 3–4) in five patients (63%) in the group with ICI reintroduction and in eighteen patients (67%) of those without ICI reintroduction (*p* = 0.581).

Three patients (75%) with severe necrosis at ICI-DILI were rechallenged and four patients (25%) were not (*p* = 0.3).

Five patients in the group with the rechallenge (63%) had received corticosteroids at ICI-DILI (Table 4). There was no difference in terms of oncological progression or death in terms of the re-introduction of ICI treatment.

## 4. Discussion

In this study, we collected the clinical, biological, histopathological and management data of 35 patients with ICI-DILI. In our cohort, patients who underwent liver biopsy had the most severe biochemical hepatitis and the most cytolytic hepatitis, which is in line with the international recommendations [7,8,9].

The liver biopsy did not fundamentally change the management of acute hepatitis: the decision to taper, suspend temporarily or definitely, and reintroduce ICI, was independent of the histological confirmation of ICI-DILI, and was mostly based on oncological disease, as already disclosed [10].

However, there was a trend in prescribing corticosteroids in patients who underwent the liver biopsy (70% vs. 40%) and this is because these patients had a more sever biological hepatitis. As already reported, the liver biopsy did not modify the time of ICI-DILI resolution [10,13].

Despite no significant difference in biochemical ICI-DILI severity according to the ICI regimen, in patients receiving anti-PD-1/anti-CTLA-4 combination therapy compared to patients receiving anti-PD-1 monotherapy, we observed a trend towards greater histological severity (30% mild-to-severe necrosis vs. 15%, respectively), and a longer time until liver-function test normalization (120 days vs. 49 days, (*p* = 0.007), respectively).

There was a trend towards a more granulomatous profile in the combination therapy group, as described by Everett et al. and De Martin et al. [14,15], without identifying, in our cohort, a pathognomonic histological profile depending on the ICI molecule [16].

Considering the evolution of ICI-DILI according to histological profile and corticosteroid treatment, we observed that the granulomatous profile tends to have a longer normalization time compared to the autoimmune and toxic profiles, even after corticosteroids, but it remains sensitive to corticosteroids [17].

According to international recommendation [7,8,9], corticosteroids were given more frequently in patients with grade 3–4 hepatitis or with severe necrosis at histology, independently of the histological profile.

Corticosteroid treatment did not influence the decision to temporarily or definitely suspend or rechallenge the ICI in this study, which was mainly based on oncological disease.

**Figure 4:**
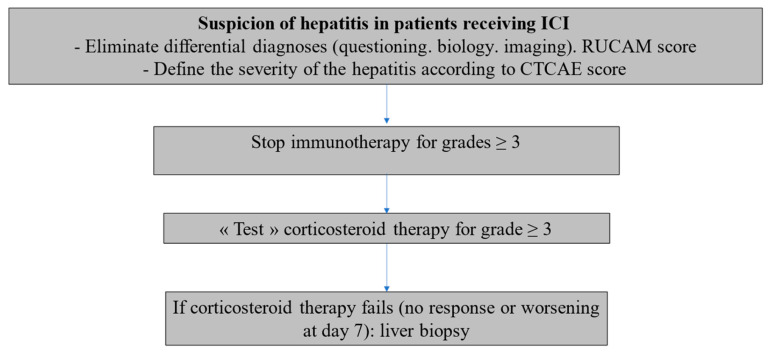
ICI-DILI: Proposal for a management algorithm.

According to our data, corticosteroids appear not to have a main impact on the evolution of ICI-DILI; we then propose a simple management algorithm in the light of the results of our study and those of the recent literature [2] (Figure 4).

In addition, we observed here that patients with a cholangitic histological profile were less responsive to corticosteroids than non-cholangitic patients, with an average time for normalization of liver function tests that was not improved in patients receiving corticosteroids compared to patients not receiving corticosteroids, as previously reported by Doherty et al. [18,19]. The biochemical cholestatic profile was associated with a histological cholangitic lesion, as already reported [20]. Finally, we observed that patients with a cholestatic biochemical profile were lower responders to corticosteroids than patients with a cytolytic profile. Patients with a cholangiocytic profile are probably those who could benefit more from UDCA treatment, as previously suggested [2], and those for whom a biopsy might be useful to rule out microscopic tumor biliary obstruction.

Our data suggest that corticosteroids could be introduced in patients with more severe ICI-DILI (CTCAE score > 3) without any improvement after ICI withdrawal or dose reduction. UDCA may be added in patients with a cholangitic profile. Patients with a predominant cholestasis, without elevated transaminases or with minimal hypertransaminasemia should be treated with UDCA alone as a first-line treatment. Corticosteroids should be added if liver tests do not improve or if they worsen. Liver biopsy could be performed in patients without improvement in a liver test under corticosteroids or UDCA. A second-line immunosuppressive treatment (mycophenolate mofetil) should be considered when liver tests do not improve under corticosteroids despite increasing doses in patients with histologically confirmed ICI-DILI.

DILI in patients treated with immunotherapy has, according to the literature, no impact on the oncological disease [21,22,23]. A Spanish group recently reported that retreatment with ICI was a feasible option after a severe immune-related hepatitis, even with the same ICI, without recurrence of liver injury, in up to 65% of patients [24]. Even if our study did not aim to assess the rechallenge of ICI, we observed that the decision of ICI resumption was taken by the oncology team, in agreement with the hepatology team, and independently of the ICI-DILI biological and histological severities.

One of the strengths of our study is the proper characterization of the ICI-related hepatotoxicity, because all patient data were evaluated by a hepatologist, according to the same exhaustive assessment, in particular with the exclusion of hepatic progression of metastatic lesions, which is one of the main causes of impaired-liver-function tests in patients treated with ICI [25]. In addition, in our cohort, the causal relationship between ICI and acute hepatitis was assessed by the RUCAM score and the severity by the CTCAE vs. 5.0 severity score.

However, this study has some limitations. First of all, it is a retrospective study associated with risks of selection bias and missing data. Moreover, it should be noted that the knowledge and recommendations regarding the management of ICI-DILI have greatly evolved during the period of our study, with increasing evidence that there is no need for corticosteroids in the treatment of severe immune-related hepatitis and there are few data addressing the usefulness of liver biopsy regarding this decision [15,26]. Another limitation is that, due to the retrospective nature of our study, it was not possible to define whether performing the biopsy delayed the management of hepatitis, as already described by Li et al. [10], even if it was performed within 15 days of ICI-DILI onset. Furthermore, this is a single-center study; the small number of patients may explain the lack of power and therefore sometimes the lack of statistical significance of our results.

To date, management of ICI-DILI remains challenging and must be patient-oriented.

This study can therefore pave the way for studies with a larger sample, which are multicenter and prospective, in order to confirm our findings, define the best management of patients with ICI-DILI and find the minimum dose of ICI with the best oncological efficacy in order to reduce the risk and the severity of ICI-DILI.

Moreover, further studies are required to elucidate the pathophysiological mechanisms and risk factors underlying ICI toxicity, to validate the predictors of resolution and recurrence after ICI reintroduction.

## 5. Conclusions

This retrospective study described how ICI-DILI has a good prognosis and is not a limiting factor for ICI use. Liver histology allows for the confirmation of the diagnosis, and could guide the ICI-DILI management, but is not essential, except for excluding other etiologies, especially in cholestatic form. In ICI-DILI, liver biopsy should not delay medical management, but may be useful for identifying patients with a cholangitic or non- defined profiles, who had a poorer response to corticosteroids. Finally, corticosteroids could be reserved/restricted to patients who did not improve after ICI withdrawal or dose reduction.

## Figures and Tables

**Table 1:** Patients’ characteristics according to liver biopsy.

Characteristics	Population	Biopsy, N (%)		*p*-Value
		Non	Yes	
Patients, *n* (%)	35 (100)	15 (43)	20 (57)	
Median age at ICI-DILI, years (IQR)	62 (48–73)	69 (45–77)	59 (49–65)	0.383
Sex, male, *n* (%)	14 (40)	6 (40)	8 (40)	0.999
Non-smoker, *n* (%)	17 (48)	8 (53)	9 (45)	0.630
Alcohol use, *n* (%)	5 (14)	1 (7)	4 (20)	0.272
Pre-existing chronic liver disease, *n* (%)	0 (0)	0 (0)	0 (0)	0.999
Underlying cancer				
Melanoma, *n* (%)	19 (54)	9 (60)	10 (50)	0.698
Lymphoma, *n* (%)	1 (3)	1 (7)	0 (0)	
Non-small-cell lung cancer, *n* (%)	10 (28)	3 (20)	7 (35)	
Other, *n* (%)	5 (14)	2 (13)	3 (15)	
Presence of liver metastases, *n* (%)	5 (14)	2 (13)	3 (15)	0.272
Other immuno-mediated side effects, *n*(%)	12 (34)	7 (47)	5 (25)	0.188
Anti-PD-1 monotherapy, *n* (%)	26 (74)	12 (80)	14 (70)	0.509
Anti-PD-1/anti-CTLA-4 combination therapy, *n* (%)	9 (25)	3 (20)	6 (30)	0.509
Previous chemotherapy, *n* (%)	15 (43)	8 (53)	7 (35)	0.285
Median of liver function tests at ICI-DILI (IQR)				
Aspartate aminotransferase (AST), U/L (IQR)	163 (68–489)	119 (51–207)	281 (80–550)	0.059
Alanine amino transferase (ALT), U/L (IQR)	344 (114–667)	154 (57–481)	642 (123–796)	0.022
Alkaline phosphatase, U/L (IQR)	144 (108–226)	133 (111–199)	171 (108–246)	0.676
Gamma-GT, U/L (IQR)	162 (71–523)	180 (124–342)	121 (53–527)	0.389
Total bilirubin (umol/L) (IQR)	8.2 (5.2–15.2)	6.4 (5.6–14.2)	8.7 (5–22.7)	0.325
Prothrombin time, (PT) (%)	80 (71–100)	100 (94–100)	83 (73.5–101)	0.383
Platelets × 10^3^/μL (IQR)	218 (190–259)	232 (193–242)	227 (195–295)	0.460
R-factor (IQR)	4.8 (2.4–9.1)	2.5 (2.2–4)	5.9 (4–14.9)	0.004
RUCAM score (IQR)	7 (6–9)	7 (5.5–9.5)	7.5 (6–8.7)	0.999
Time until liver function tests normalization, days (IQR)	60 (40–120)	49 (37–97)	60 (42–135)	0.205
ICI discontinuation, *n* (%)	32 (91)	13 (87)	19 (95)	0.390
Reduction of ICI dosage, *n* (%)	4 (11)	2 (13)	2 (10)	0.776
Corticosteroid therapy, *n* (%)	20 (57)	6 (40)	14 (70)	0.080
Rechallenge N (%) yes				
same ICI	4 (11)	2 (13)	2 (10)	0.762
other ICI	4 (11)	2 (13)	2 (10)	0.762
non	27 (77)	11 (73)	16 (80)	

**Table 2:** Patients’ characteristics according to ICI regimen.

Characteristics		Population (35)	Anti-PD-1 Monotherapy (26)	Anti-PD-1 + Anti-CTLA-4 Combination Therapy (9)	*p*-Value
Median age at ICI-DILI, years (IQR)		62 (48–73)	65 (55–73)	48 (45–62)	0.043
Sex, male, *n* (%)		14 (40)	10 (38)	4 (44)	0.756
Underlying cancer	Melanoma, *n* (%)	19 (54)	10 (38.5)	9 (100)	0.003
	Lymphoma, *n* (%)	1 (3)	1 (4)	0 (0)	
	Non-small-cell lung cancer, *n* (%)	10 (28)	10 (38.5)	0 (0)	
	Other, *n* (%)	5 (14)	5 (19)	0 (0)	
Previous chemotherapy, *n* (%)		15 (43)	13 (50)	2 (22)	0.153
Treatment line, *n* (IQR)		1 (1–2)	1 (1–2)	1 (1–2)	0.555
Number of ICI cycles, *n* (IQR)		3 (1–4)	2.5 (1–4.25)	3 (2.5–4)	0.476
Other IRAES, *n* (%)		12 (34)	8 (31)	4 (44)	0.463
AST, U/L (IQR)		163 (75–483)	156 (64–490)	194 (96–428)	0.942
ALT, U/L (IQR)		300 (115–665)	344 (115–665)	285 (114–891)	0.961
ALP, U/L (IQR)		152 (108–223)	172 (112–250)	126 (71–166)	0.104
Gamma-GT, U/L (IQR)		147 (76–488)	196 (94–526)	115 (32–154)	0.104
Total bilirubin, (umol/L) (IQR)		8.2 (5.2–15.2)	7.6 (5–19.2)	9.8 (6.4–10.6)	0.438
Prothrombin time, (PT) (%)		100 (78–100)	98 (72–100)	100 (81–101)	0.524
Platelets × 10^3^/μL, (IQR)		231 (199–255)	231 (193–251)	226 (193–281)	0.925
R-factor (IQR)		4.8 (2.4–9.1)	4.35 (2.28–6.68)	5.9 (2.5–21.5)	0.184
RUCAM score (IQR)		7 (6–9)	8 (6.7–9)	6 (6–8)	0.130
Auto antibodies, *n* (%)	FAN > 1/80 ASMA > 1/80	19 (54) 2 (6)	14 (54) 2 (8)	5 (57) 0 (0)	0.956 0.398
Corticosteroid therapy, *n*(%)		20 (57)	13 (50)	7 (78)	0.153
Time until liver function tests normalization, days (IQR)		60 (40–120)	49 (37–67)	120 (75–150)	0.007
Histological pattern, *n* (%), available for 20 patients	Toxic	6 (30)	5 (36)	1 (17)	0.406
	Autoimmune	1 (5)	0 (0)	1 (17)	0.141
	Granulomatous	6 (30)	3 (21)	3 (50)	0.253
	Mixed (toxic + autoimmune)	1 (5)	1 (7)	0 (0)	0.497
	None	6 (30)	5 (36)	1 (17)	0.355
Histological pattern, *n* (%)	Minimal necrosis	10 (50)	9 (45)	1 (5)	0.057
	Mild necrosis	2 (10)	0 (0)	2 (10)	0.026
	Severe necrosis	7 (35)	3 (15)	4 (20)	0.058
ICI discontinuation, *n* (%)		32 (91)	23 (88)	9 (100)	0.294
ICI dose reduction, *n* (%)		4 (11)	1 (4)	3 (33)	0.021
ICI rechallenge, *n* (%)	Yes Same ICI Other ICI No	4 (11) 4 (11) 27 (77)	2 (8) 3 (11) 21 (80)	2 (22) 1 (11) 6 (77)	0.244 0.073

**Table 3:** Patients’ characteristics and management according to corticosteroids introduction.

Characteristics		With Corticosteroids (20)	Without Corticosteroids (15)	*p*-Value
Patient profile:				
Type of immunotherapy, *n* (%)	Anti-PD-1 monotherapy	13 (65)	13 (87)	0.153
	Anti-PD-1 + anti-CTLA-4 combination therapy	7 (35)	2 (13)	0.153
Biochemical severity (ALT), *n* (%)	Grade 1	3 (15)	2 (13)	0.095
	Grade 2	2 (10)	5 (33)	
	Grade 3	6 (30)	6 (40)	
	Grade 4	9 (45)	2 (13)	
Histological severity (necrosis), *n* (%)	minimal	5 (25)	5 (33)	0.057
	mild	2 (10)	0 (0)	0.342
	severe	7 (35)	0 (0)	0.036
Histology (yes), *n* (%)		14 (70)	6 (40)	0.080
Histological pattern, *n* (%)	Toxic	5 (25)	1 (7)	0.406
	Autoimmune	1 (5)	0 (0)	0.497
	Granulomatous	4 (20)	2 (13)	0.913
	Mixed Non	1 (5) 3 (15)	0 (0) 3 (20)	0.497 0.253
Characteristics of care:				
ICI discontinuation, *n* (%)		19 (95)	13 (87)	0.390
ICI rechallenge (same or other ICI), *n* (%)		4 (25) 1 (5)	3 (20) 0 (0)	0.070 0.174
Time until improvement in liver function, *n* (%)		90 (60–120)	40 (30–57)	0.015

**Table 4:** Patients’ characteristics according to ICI reintroduction after ICI-DILI.

Characteristics	ICI Retreated Patients (8)	Non ICI-Retreated Patients (27)	*p*-Value
Biopsed, *n* (%)	4 (50)	16 (59)	0.647
Anti-PD-1 monotherapy, *n* (%)	5 (63)	21 (78)	0.392
Anti-PD-1/anti-CTLA-4 combination therapy, *n* (%)	3 (38)	6 (22)	0.392
Biochemical severity, grade 3–4 ALT at ICI-DILI, *n* (%)	5 (63)	18 (67)	0.378
Histological severity at ICI-DILI, *n* (%)	3 (38)	4 (15)	0.068
Corticosteroids, *n* (%)	5 (63)	15 (56)	0.731
1-month oncological disease progression, *n* (%)	4 (50)	9 (33)	0.598
3-month oncological disease progression, *n* (%)	4 (50)	8 (30)	0.454
6-month oncological disease progression, *n* (%)	2 (25)	3 (11)	0.433
12-month oncological disease progression, *n* (%)	2 (25)	4 (15)	0.703
Death	4 (50)	12 (44)	0.923

## Data Availability

Data sharing: subject to agreement.

## Supplementary Materials

The following supporting information can be downloaded at https://www.mdpi.com/article/10.3390/jcm12113751/s1, Table S1: Raw histological characteristics according to ICI regimen.
