# Supplementary material for: Histological Characteristics and Management of Hepatitis on Immune Checkpoint Inhibitors: A Retrospective Descriptive Study
Source: J Clin Med. 2023 May 29;12(11):3751. doi: 10.3390/jcm12113751 (PMC10253512; doi:10.3390/jcm12113751)
Supplement: Supplementary file 1 [file jcm-12-03751-s001.zip › jcm-2377416-supplementary.pdf]

Journal of
Clinical Medicine

MDPI

Table S1. Raw histological characteristics according to ICI regimen.

Histological characteristics		Populatio n (20)	Anti-PD-1 monotherap y (14)	Anti-PD-1 + anti-CTLA-4 combinatio n therapy (6)	p-value
Granuloma		6 (30)	3 (21)	3 (50)	0.879
Plasma cells		2 (10)	1 (7)	1 (17)	0.125
Eosinophils		9 (45)	6 (43)	3 (50)	0.814
Steatosis		5 (25)	4 (28)	1 (17)	0.708
Fibrosis		5 (25)	2 (14)	3 (50)	0.077
Endothelitis		6 (30)	3 (21)	3 (50)	0.265
Cholangitis		9 (45)	5 (36)	4 (67)	0.509
Ductopenia		1 (5)	0 (0)	1 (17)	0.269
Sinusoidal dilatation		2 (10)	1 (7)	1 (17)	0.939
Necrosis	minimal	10 (50)	9 (64)	1 (17)	0.057
	mild	2 (10)	0 (0)	2 (33)	0.026
	severe	7 (35)	3 (21)	4 (67)	0.058
Portal involvement		19 (95)	13 (93)	6 (100)	0.365
Lobular involvement		19 (95)	13 (93)	6 (100)	0.365
Tumor involvement		1 (5)	0 (0)	1 (17)	0.269
